# A New Species of *Orthosyntexis* (Hymenoptera: Anaxyelidae) from Mid-Cretaceous Burmese Amber

**DOI:** 10.3390/insects16101039

**Published:** 2025-10-09

**Authors:** Xiao Li, Gengyun Niu, Meicai Wei

**Affiliations:** Laboratory of Insect Systematics and Evolutionary Biology, College of Life Sciences, Jiangxi Normal University, Nanchang 330022, China

**Keywords:** *Orthosyntexis*, Kachin amber, Syntexinae, Symphyta, new species

## Abstract

**Simple Summary:**

Sawflies of the family Anaxyelidae are represented today by a single rare species in western North America, which lays its eggs in fire-damaged conifers. Fossils, however, show that these insects were far more diverse during the age of dinosaurs. In this study, we describe a new species from 99-million-year-old Burmese amber, providing fresh evidence of their Cretaceous diversity. We also re-examined an older fossil, *Kempendaja jacutensis*, and confirmed that an earlier interpretation of its wing venation was likely an error. Finally, we found that the presence or absence of a small crossvein in the forewing is consistently associated with differences in the hind wing: a pattern that may help future researchers untangle evolutionary relationships.

**Abstract:**

Anaxyelidae, a relict lineage of sawflies, are represented by a single extant species today but displayed remarkable Mesozoic diversity. Here, we describe the *Orthosyntexis mascula* sp. nov. from mid-Cretaceous Burmese amber. The new species can be readily distinguished by its forewing, with a normally sized, uniformly sclerotized pterostigma; 1-Rs shorter than 1-M; cell 1M more than twice as long as wide; absence of 1r-rs; 1-Cu, distinctly shorter than 2-Cu; 3-Cu shorter than 4-Cu; 2m-cu shorter than 1m-cu; and 3rs-m twice as short as 4-M. In the hind wing, abscissa 2-M+Cu present, 1-M shorter than 2-M, crossvein m-cu absent, and cell R1 closed. Mesotibia with two apical spurs. Examination of high-resolution photographs of *Kempendaja jacutensis* enables a revised interpretation of its venation, confirming its placement in Anaxyelinae. Comparative analysis of syntexine taxa further reveals that variation in the forewing crossvein 1r-rs consistently corresponds with hind wing venation, suggesting that multiple evolutionary trajectories may have existed within Syntexinae. These findings not only expand the known diversity of Cretaceous Anaxyelidae but also provide new evidence for reconstructing the evolutionary history and internal diversification of Anaxyelidae.

## 1. Introduction

Anaxyelidae are a highly distinctive group of sawflies, currently represented by only a single extant species, *Syntexis libocedrii* [1], which occurs in western North America and is typically associated with fire-damaged conifers [2,3,4]. In contrast to its limited modern representation, Anaxyelidae have a rich fossil record, offering significant insights into their diversity during the Jurassic and Cretaceous periods, providing key evidence for understanding the early evolution of Siricoidea. According to the recent fossil catalog by Li et al. (2024), the family comprises 25 fossil genera and 54 species [5].

Currently, Anaxyelidae are divided into two subfamilies: Anaxyelinae and Syntexinae [4,6,7,8]. Rosse-Guillevic et al. (2023) proposed five diagnostic characters for distinguishing Syntexinae from Anaxyelinae [9]. Among these, the position of the Rs+M bifurcation relative to cell 1M is the most decisive, with Syntexinae typically having the fork placed well beyond the middle of cell 1m-cu, or even distal to crossvein 1m-cu. The remaining characters, including the proportions and apical shape of cell 2r, the position of vein 2r-rs on the pterostigma, and the apical shape of cell 3r, also provide useful distinctions, although exceptions occur [9,10]. Most known species of Syntexinae, with the exception of *Daosyntexis and Karasyntexis*, are from the Cretaceous, and, to date, 13 genera and 19 species have been described within this subfamily [5,9,10,11,12,13,14,15,16,17,18,19].

The taxonomic placement of certain anaxyelid genera has been debated. *Kempendaja* is a representative case: it was originally assigned to its own subfamily, Kempendajinae, based on a supposed longitudinal Sc vein [7,8,11]. Later phylogenetic work (e.g., Gao et al., 2021) supported merging Kempendajinae into Anaxyelinae [15], and Kopylov (2025) further argued that the earlier interpretation of a longitudinal Sc vein in *Kempendaja* was mistaken, likely resulting from taphonomic distortion [10]. Here, new high-resolution photographs confirm the absence of Sc in *Kempendaja*, thereby reinforcing its placement within Anaxyelinae.

Within Syntexinae, a diverse subfamily of Anaxyelidae, forewing venation, particularly the presence or absence of the crossvein 1r-rs, shows striking variation among genera [18]. Yet, the evolutionary and taxonomic implications of this character remain insufficiently explored, and potential correlations between fore- and hind wing venation have received little attention. In this study, we describe a new species of *Orthosyntexis* from mid-Cretaceous Burmese amber and reassess the venation of *Kempendaja jacutensis* to clarify its taxonomic placement. We also investigate correspondences between fore- and hind wing venation within Syntexinae, thereby providing new insights into the evolutionary history of Anaxyelidae.

## 2. Materials and Methods

Specimens were examined under a Motic SMZ-171 stereomicroscope (Motic, Xiamen, China). Adult images were captured using a Leica Z16 APO microscope (Leica Microsystems, Wetzlar, Germany), then focus-stacked with Helicon Focus (HeliconSoft, Kharkiv, Ukraine), and further processed using Adobe Photoshop CS23. Holotypes of the new species are deposited in the Asian Sawfly Museum, Nanchang, China (ASMN). The specimen was collected from Kachin (Hukawng Valley) in northern Myanmar, deposits of which were dated at 99 Mya [20,21].

In this study, we followed the modified wing venation nomenclature proposed by Goulet and Huber [22]. The vein symbols used in our analysis include major longitudinal veins (e.g., Sc, R, Rs, Rs+M, M, Cu, and M+Cu). Sections of these veins are labeled as 1-Rs, 1-M, etc., while crossveins are designated as 1r-rs, 2r-rs, 2r-m, and similar. We also use standardized terms for wing cells (e.g., 1R, 2Rs, 1M) to ensure consistency and clarity in interpretation.

## 3. Results

**Systematics Paleontology**

**Family Anaxyelidae Martynov, 1925**

**Subfamily Syntexinae Benson, 1935**

**Genus *Orthosyntexis* J. Gao, Engel, Shih, and T. Gao, 2021**

**Type species.** *Orthosyntexis elegans* J. Gao, Engel, Shih, and T. Gao, 2021

**Species included.** *Orthosyntexis elegans* J. Gao, Engel, Shih, and T. Gao, 2021; *Orthosytexis cretacicus* Zheng, 2025; *Orthosyntexis thani* J. Gao, Engel, Shih, and T. Gao, 2021; *Orthosyntexis mascula* Li and Wei, sp. nov.

**Revised diagnosis.** Pronotum short, having a prominent anterior notch and hind margin, with a median longitudinal furrow dorsally. Forewing with pterostigma not enlarged, uniformly sclerotized and of normal width; 1-Rs slightly shorter than or subequal to 1-M; cell 1M length to width ratio slightly more than 2; Rs+M bifurcated after 1m-cu, 2-Rs+M short; cell 2M hexagonal, with a length slightly greater than its width (ratio is approximately 1.2:1); 1-Cu shorter than 2-Cu; 3-Cu slightly shorter than or equal to 4-Cu; 2m-cu 1.5× shorter than 1m-cu. Hind wing with cell R1 closed, abscissa 2-M+Cu present; 1-M shorter than 2-M; cu-a intersects with 2-M+Cu. Mesotibia with two apical spurs.

***Orthosyntexis mascula* Li and Wei, sp. nov.**

**LSID:** http://zoobank.org/urn:lsid:zoobank.org:act:087B79C7-975E-4399-AAC8-3D0703C9A662 (accessed on 6 October 2025).

**Etymology.** The species name is derived from the Latin *masculus* (“male”), here modified to the feminine form *mascula* to agree in gender with the generic name *Orthosyntexis*.

Locality and horizon. The amber specimen was collected from Kachin (Hukawng Valley) in northern Myanmar and is dated at 98.79 ± 0.62 Mya [20,21].

**Diagnosis.** Compound eyes surrounded by distinct white margins. In the forewing, 1-Rs perpendicular to R and approximately equal in length to 1-M, meeting it at a right angle; 2r-rs twice as long as the width of pterostigma; 3rs-m perpendicular to 4-M; and 2-Cu approximately 1.3 times the length of 1-Cu. Legs generally pale, with dark markings on the mesofemur and metatibia.

**Figure 1:**
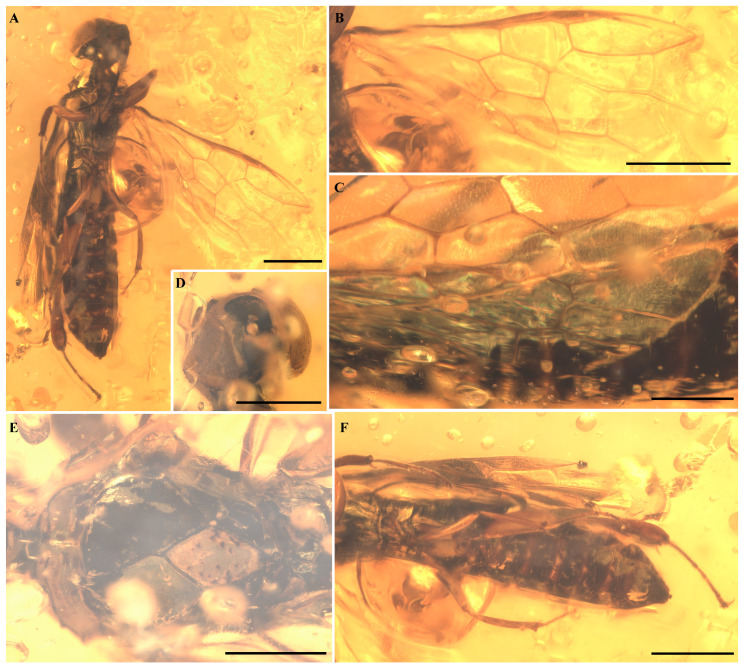
Photographs of *Orthosyntexis mascula* sp. nov. (**A**) Habitus in ventral view. (**B**) Forewing. (**C**) Hind wing. (**D**) Head in dorsal view. (**E**) Detailed view of the mesosoma. (**F**) Detailed view of legs. Scale bar: 1 mm in (**A**,**B**,**F**); 0.5 mm in (**C**–**E**).

**Figure 2:**
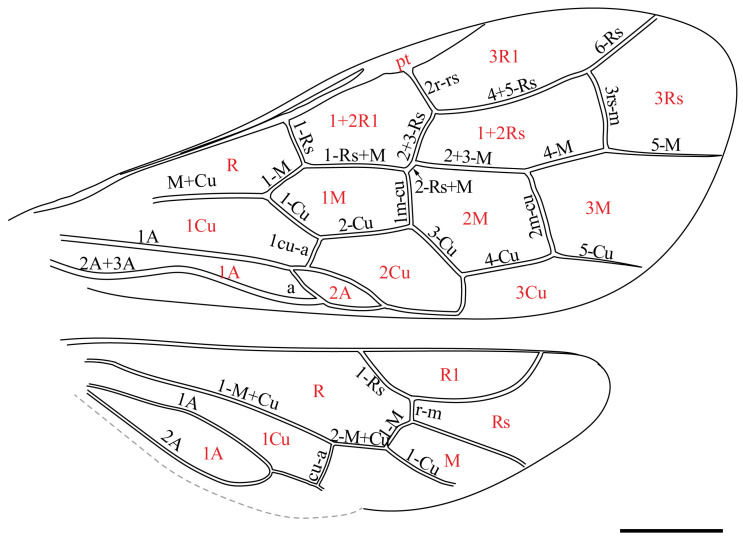
*Orthosyntexis mascula* sp. nov., holotype. Interpretative line drawing of the wing’s venation, with names of veins and cells labeled. Scale bar: 0.5 mm.

**Holotype.** Male. ASMN-FA-2025-01, deposited in the Asian Sawfly Museum, Nanchang, China (ASMN) (Figure 1 and Figure 2)

**Measurements.** Total body length 6.0 mm (lateral view); antenna length 1.7 mm (ventral view); forewing approximately 3.6 mm long and 1.5 mm wide at maximum; hind wing about 3.0 mm in length.

**Head.** Moderately large, as wide as the thorax (1.0 mm wide, 0.6 mm long); black in coloration, with white margins encircling the compound eyes (Figure 1D). Compound eyes large and hemispherical. Antennae elongate, comprising approximately 16 segments.

**Thorax.** Broad, with width across tegulae 1.3 mm. Pronotum short, with a distinct anterior notch; dorsally bearing a median longitudinal furrow. Mesoscutum with a strongly impressed median longitudinal sulcus and notauli; mesoscutellum rhombic, tapering to a sharp apex. Proportions of prescutum:sulcus:mesoscutum–mesoscutellar sulcus: mesoscutellum = 4.4:1:7.5. Notauli terminate near the mesoscutellum (Figure 1E).

**Legs.** Slender; profemur and mesofemur longer than corresponding tibiae; etafemur shorter than metatibia (metafemur 1.1 mm; metatibia 1.3 mm, thickened subapically). Basal portions of profemur, protibia, and tarsomeres IV–V dark; other segments pale. Middle leg entirely pale with a dark spot on outer surface of mesofemur. Metafemur and metatibia pale, with apical part of metatibia black; metatarsus dark. One apical spur present on protibia and metatibia; mesotibia with two apical spurs (Figure 1F). Basitarsus elongate but shorter than combined length of remaining tarsomeres; tarsomere V elongate but shorter than basitarsus. Metatarsal claws long, apically curved, each with a distinct preapical tooth.

**Abdomen.** Slightly narrower than mesothorax; first tergite split medially. Genitalia not visible.

**Forewing.** Forewing with dense microtrichia but no coloration pattern except a slightly darkened costal area. C and R veins thick, and costal area narrower than C and R widths. Pterostigma completely sclerotized; Sc vein absent; 1r-rs and 2rs-m absent; 2r-rs issuing from pterostigma at its basal 1/3; 1-Rs short and sub-vertical to R, about as long as 1-M, meeting 1-M at right angle. 2-Rs+M distinct but short, shorter than 2r-rs, 2r-rs slightly proclival. 2+3-M 1.4× as long as 4-M. Cell 1M pentagonal, about 2.0× as long as wide; cell 2M hexagonal, about as long as wide. 2-Cu about 1.3× as long as 1-Cu. 1cu-a strongly reclival, nearly 0.43× as long as 1-Cu. 1m-cu 0.74× as long as 3-Cu, 3-Cu shorter than 4-Cu. 2m-cu nearly 1.1× as long as 4-M and 1.6× as long as 1m-cu. 1A nearly straight, curved after 1cu-a toward wing posterior margin. Internal crossvein short and moderately oblique. 2A+3A sinuate and in part far from the posterior margin of the wing. Anal cells ordinary (not narrow) (Figure 1B and Figure 2).

**Hind wing.** The hamuli are not visible. Sc vein absent. Cell R1 closed at the wing anterior margin. 1-Rs longer than 1-M, r-m with an angle at 2-Rs and shorter than 1-Rs, 1-M straight except bend slightly before meeting point with r-m; m-cu absent; 2-M+Cu present, slightly longer than cu-a; 1-Cu well developed, nearly straight, reaching wing margin; cu-a straight; 1-A and 2-A meeting before cu-a at distance slightly superior to length of cu-a (Figure 1C and Figure 2).

**Remarks.** The new species differs from other congeners primarily in venational features. In *O. mascula* sp. nov., vein 2r-rs distinctly longer than the width of the pterostigma, vein 1-Cu conspicuously longer than 1-M, with 1-M subequal to 1-Rs. In contrast, in *O. thanti* and *O. elegans*, 2r-rs approximately equal to the width of the pterostigma, 1-Cu nearly as long as 1-M, and 1-M clearly longer than 1-Rs. *O. thanti* further differs in having 1-Rs slightly proclival relative to R, with crossveins 3rs-m and 2m-cu not perpendicular to M [15].

Furthermore, compared with the recently described male of *O. cretacicus*, which has a slender body with a total length of 8.09 mm, our new species is distinctly smaller (6.0 mm). In *O. cretacicus*, the longitudinal sulcus between the notauli and the mesoscuto–mesoscutellar sulcus considerably longer, the forewing 1-Rs is proclival and meets R at an angle of about 60°, cell 1M narrow and elongate, vein 2-Cu more than three times the length of 1-Cu, and vein cu-a meets the branching point of 1A, all of which clearly distinguish it from *O. mascula* [19].

**Subfamily Anaxyelinae Martynov 1925**

**Genus *Kempendaja* Rasnitsyn, 1968**

***Kempendaja jacutensis* Rasnitsyn, 1968**

**Original Description.** Rasnitsyn, A.P. 1968. Hoвые мезoзoйские пилильщики (Hymenoptera, Symphyta) [New Mesozoic sawflies (Hymenoptera, Symphyta)]. Pp. 190–236. *In*: Rohdendorf, B.B. (ed.) Юрскoй Hасекoмые Каратау [Jurassic insects of Karatau]. Izdatelstvo “Nauka”, Moscow, 252 pp., 25 pls. (in Russian) [7].

**Material Examined.** The description is based on high-resolution photographs of the fossil specimens provided by the original author, Rasnitsyn, A.P.

**Figure 3:**
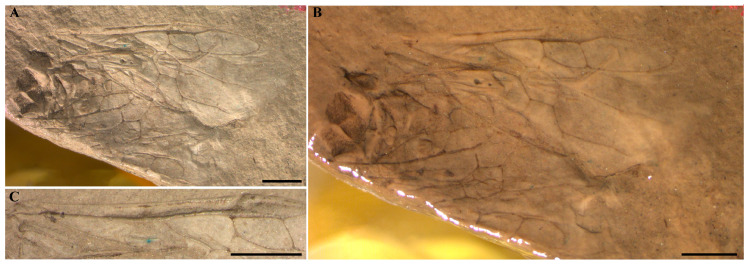
High-resolution photograph of the fossil specimen of *Kempendaja jacutensis* Rasnitsyn, 1968. (**A**) Forewing showing preserved venation details. (**B**) Forewing photographed under different illumination. (**C**) Close-up view showing the absence of Sc vein. Photo credit: Rasnitsyn, A.P. Scale bar: 1 mm.

**Revised Description.** The forewing with C and R veins thick, and costal area wider than C and R widths. Pterostigma desclerotized; Sc vein absent (Figure 3C); 2r-rs 1.8× as long as 1r-rs, 2r-rs issuing from pterostigma slightly beyond its midpoint; 1-Rs long and proclivate to R, about 3× as long as 1-M, meeting 1-M at right angle. Rs+M bifurcated before reaching the middle of cell 1M, 1-M longer than Rs+M; 2-M 2.5× as long as Rs+M; cell 1M hexagonal, about 1.4× as long as wide; cell 2M pentagonal, about 1.5× as wide. 3rs-m 2× as long as 5-M, 2m-cu 1.8× as long as 5-M. 2-Cu slightly longer than 1-Cu. cu-a perpendicular to 1A, located in the middle of anal cell (Figure 3A,B).

## 4. Discussion

### 4.1. Taxonomic Assessment of the New Species

The new species *Orthosyntexis mascula* sp. nov. can be confidently assigned to the family Anaxyelidae based on its venational ground plan, and notably, the absence of the forewing crossvein 2rs-m and the presence of a single r-m in the hind wing. Within the subfamily Syntexinae, its diagnostic combination of characters, notably the Rs+M fork positioned beyond 1m-cu, a condition typical of syntexines [9,10], provides strong support for this placement. Additional traits, including a perpendicular 1-Rs nearly equal in length to 1-M, the absence of 1r-rs, a hexagonal cell 2M, a closed R1 cell in the hind wing with a distinct 2-M+Cu, and the presence of two apical spurs on the mesotibia, further corroborate its assignment to *Orthosyntexis* [15,19].

It is noteworthy that all previously described species of *Orthosyntexis* were known only from females, with body lengths ranging from 8.25 to 8.61 mm [15]. More recently, a male of *O. cretacicus* was described, with a body length of 8.09 mm, which is comparable to that of the known females [10]. By contrast, the new species, *O. mascula* sp. nov., provides an additional male specimen with a distinctly smaller body length (6.0 mm), making it the smallest known representative of the genus to date. In the extant *Syntexis libocedrii*, females measure 8–16 mm in length, whereas males are smaller at 6–12 mm: on average, about two-thirds of the size of females [1,10]. The small size of *O. mascula* thus appears to be consistent with this pattern, but the case of *O. cretacicus* suggests that such correlations may not be universal. With so few fossil specimens available, no firm conclusions can yet be drawn regarding sexual size dimorphism in extinct *Orthosyntexis*.

A defining feature of *Orthosyntexis* is the absence of the forewing 1r-rs, a trait shared with several other genera of Syntexinae, including *Curvitexis*, *Deresyntexis*, *Eosyntexis*, *Paraxiphydria*, and *Sclerosyntexis*. Among the distinguishing features, one notable difference is that whereas *Curvitexis*, *Deresyntexis*, and *Eosyntexis* exhibit a proclival 1-Rs, *Orthosyntexis* possesses a nearly perpendicular 1-Rs, relative to R [5,9,14,18,23,24,25]. In the hind wing, a further contrast is seen: *Paraxiphydria* and *Sclerosyntexis* show a reduction where 2-M+Cu and 1-M are fused due to the loss of free Cu [16,26], whereas in *Orthosyntexis*, the cu-a meets M+Cu well before its end, leaving a free and elongated 2-M+Cu [15].

### 4.2. Notes on Anaxyelinae: *Kempendaja jacutensis*

Our re-examination of *Kempendaja jacutensis* using high-resolution photographs revealed that the Sc vein is absent, contrary to earlier interpretations. Rasnitsyn (1968) originally described *Kempendaja* within Anaxyelinae, with no mention of a longitudinal Sc vein in the forewing [7]. Later, however, Rasnitsyn (1980) erected a separate subfamily, Kempendajinae, based on the supposed presence of a longitudinal Sc [8]. Following this, Kopylov (2019) described *Mangus* in Kempendajinae and, relying on Rasnitsyn’s interpretation, also noted a longitudinal Sc in *Kempendaja*, although the holotype had not been carefully re-examined [11]. Gao et al. (2021) subsequently re-evaluated the venation and explicitly stated that *Kempendaja* lacks a longitudinal Sc [15]. This conclusion initially surprised both Kopylov and Rasnitsyn, who then re-checked the material and confirmed that the earlier interpretation of a longitudinal Sc was mistaken. As reported by Kopylov and Rasnitsyn (2025), the supposed Sc in *Kempendaja* most likely represents an optical artifact produced by taphonomic distortion of the wing, and the earlier establishment of Kempendajinae was therefore unjustified [10].

Our observations are consistent with this interpretation: *Kempendaja* lacks a true Sc, firmly supporting its placement within Anaxyelinae. In contrast, *Mangus magnus* genuinely retains a well-developed and longitudinally bifurcated Sc vein, a plesiomorphic condition not observed in other anaxyelines [11]. Apart from this primitive venational trait, the overall morphology of *M. magnus* conforms well to the diagnostic features of Anaxyelinae [15]. Consequently, *Mangus magnus* is also regarded as a member of Anaxyelinae, and we infer that it likely represents an early-diverging, relatively basal lineage within the subfamily.

### 4.3. Forewing and Hind Wing Venation Patterns Within Syntexinae

Syntexinae are currently treated as a monophyletic group, primarily distinguished from Anaxyelinae by the position of the Rs+M fork beyond the middle of cell 1M, and this character appears stable across the subfamily [9,10]. Building on this established framework, our examination of described syntexine species reveals an additional, noteworthy pattern: variation in the presence of the forewing 1r-rs consistently corresponds with hind wing venation.

Nevertheless, hind wings are not preserved, or remain unknown, in more than half of the described genera. Consequently, the correlations presented below are established only for taxa with sufficiently preserved hind wings, while in many others, the relevant structures cannot yet be assessed. These correspondences should therefore be regarded as preliminary observations, rather than universally applicable features across Syntexinae.

**Figure 4:**
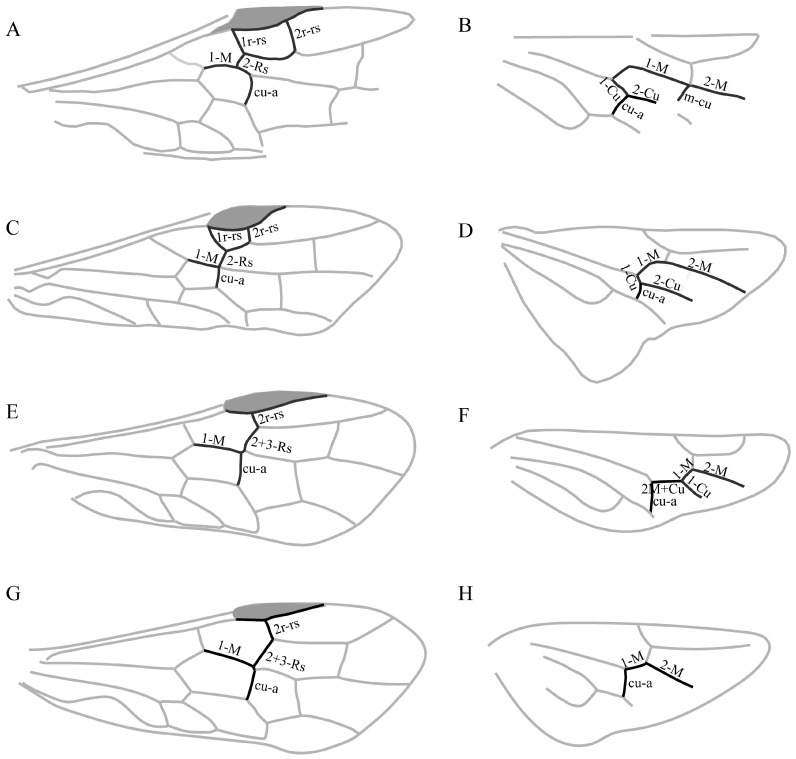
Wing venation patterns in Anaxyelidae: (**A**,**B**) *Anaxyela gracilis* (Anaxyelinae), retaining forewing 1r-rs and showing a plesiomorphic hind wing; (**C**,**D**) *Syntexis libocedrii* (Syntexinae), also with 1r-rs retained and a corresponding basal hind wing pattern; (**E**,**F**) *Orthosyntexis elegans* (Syntexinae), lacking forewing 1r-rs and displaying a free, elongated 2-M+Cu; (**G**,**H**) *Paraxiphydria resinata* (Syntexinae), lacking forewing 1r-rs and showing fusion of 2-M+Cu and 1-M.

In the forewing, 1r-rs occurs in two distinct states. It is retained in *Curiosyntexis*, *Daosyntexis*, *Dolichosyntexis*, *Hanguksyntexis*, *Hemisyntexis*, *Karasyntexis*, *Parasyntexis*, and *Syntexis* (Figure 4C,D). In *Curiosyntexis*, however, 1r-rs only weakly developed, and the hind wing is poorly preserved in the available material, so the comparison is of limited relevance [11]. These taxa exhibit hind wings resembling those of basal Anaxyelinae (Figure 4A,B): the M between M+Cu and r-m distinctly curved, with the branches of M+Cu running in parallel after bifurcation. The cu-a meets Cu near the end of M+Cu, so that 1-Cu shorter than half of cu-a [1,11,17]. The r-m proximally positioned, and the m-cu absent, except in *Parasyntexis khasurtensis* [11].

By contrast, 1r-rs entirely absent in *Curvitexis*, *Deresyntexis*, *Eosyntexis*, *Orthosyntexis*, *Paraxiphydria*, and *Sclerosyntexis* (Figure 4E–H). The hind wing further reveals two venational variants:

(1) Loss of 1r-rs with free 2-M+Cu (Figure 4E,F). In *Orthosyntexis* and *Curvitexis*, the hind wing shows cu-a joining M+Cu considerably proximal to its distal end, leaving a free and elongate 2-M+Cu [9,15,18].
(2) Loss of 1r-rs with fused 2-M+Cu and 1-M (Figure 4G,H). In *Paraxiphydria* and *Sclerosyntexis*, 2-M+Cu and 1-M completely fused due to the loss of free Cu, producing a highly derived venational condition [16,26].

Both hind wing variants share the loss of forewing 1r-rs and may represent divergent modifications of a single evolutionary trend, rather than independent types. A further caveat is that, among anaxyelids lacking 1r-rs, hind wings are currently documented only from Burmese amber. During the mid-Cretaceous, Burma represented an isolated island in the Tethys Ocean, where evolutionary trajectories were strongly shaped by insular conditions. It is therefore possible that some venational modifications observed in Burmese syntexines reflect local, lineage-specific adaptations, rather than the evolutionary history of the subfamily as a whole. This biogeographic constraint underscores the need for additional well-preserved fossils from other localities to test the generality of these patterns.

Taken together, the correspondence between forewing and hind wing venation patterns observed across Syntexinae suggests that these features may hold more than purely descriptive value. Although our present study is based on a limited number of taxa and relies on morphological comparisons alone [9,10,15], the repeated association of forewing 1r-rs with specific hind wing configurations suggests that venational disparity could provide informative characters for future systematic analyses. At this stage, these observations are best regarded as a working hypothesis, rather than a resolved framework for internal relationships within the subfamily. Formal phylogenetic studies, ideally incorporating additional fossil discoveries and a broader set of characters [15], will be necessary to determine whether the venational patterns documented here reflect independent reductions, adaptive shifts, or deeper evolutionary trajectories.

In a broader evolutionary context, Kopylov (2025) proposed that the decline in Anaxyelidae during the Late Cretaceous period coincided with the global rise in angiosperms [10]. This study provides an additional perspective on this view: while ecological changes may indeed have contributed to their decline, the morphological disparity within Syntexinae points to substantial internal diversification preceding their disappearance. Whether venational reductions represent adaptive simplification linked to ecological shifts or independent vein-loss trends remains unresolved. Future fossil discoveries, particularly from Jurassic to Cretaceous transitional deposits, together with functional and phylogenetic studies, will be crucial to evaluate these possibilities. Such work will also help place syntexine evolution within the broader context of Cretaceous insect–plant interactions.

## 5. Conclusions

The description of *Orthosyntexis mascula* sp. nov. from mid-Cretaceous Kachin amber expands the known diversity of Syntexinae and provides additional evidence for the morphological disparity within Anaxyelidae. Diagnostic venational and leg features firmly support its assignment to *Orthosyntexis*, while comparative analysis highlights the absence of forewing 1r-rs as a key trait linking this genus with several other syntexines. Our re-examination of *Kempendaja jacutensis* confirms the absence of Sc, thereby supporting its placement within Anaxyelinae and clarifying a long-standing misinterpretation of venation.

Taken together, these findings underscore the taxonomic value of wing venation in distinguishing lineages and reveal consistent correspondences between forewing and hind wing characters within Syntexinae. Such patterns suggest the presence of multiple evolutionary trajectories in the subfamily, although formal phylogenetic analyses will be required to test this hypothesis. Future discoveries of well-preserved fossils, particularly from transitional Jurassic–Cretaceous deposits, will be critical for further resolving the evolutionary history of Anaxyelidae. This highlights venational disparity as a potentially informative character system for understanding syntexine diversification, a hypothesis that requires further fossil evidence and phylogenetic testing.

## Data Availability

No new data were created or analyzed in this study. Data sharing is not applicable to this article.
